# Inhibitory effects of astaxanthin on postovulatory porcine oocyte aging in vitro

**DOI:** 10.1038/s41598-020-77359-6

**Published:** 2020-11-19

**Authors:** Bao-Yu Jia, De-Cai Xiang, Qing-Yong Shao, Bin Zhang, Shao-Na Liu, Qiong-Hua Hong, Guo-Bo Quan, Guo-Quan Wu

**Affiliations:** 1 College of Veterinary Medicine, Yunnan Agricultural University, Kunming, 650201 Yunnan China; 2 Yunnan Provincial Engineering Laboratory of Animal Genetic Resource Conservation and Germplasm Enhancement, Yunnan Animal Science and Veterinary Institute, Kunming, 650224 Yunnan China

**Keywords:** Ageing, Embryology, Molecular biology

## Abstract

Mammalian oocytes represent impaired quality after undergoing a process of postovulatory aging, which can be alleviated through various effective ways such as reagent treatment. Accumulating evidences have revealed the beneficial effects of astaxanthin (Ax) as a potential antioxidant on reproductive biology. Here, porcine matured oocytes were used as a model to explore whether Ax supplement can protect against oocyte aging in vitro and the underlying mechanism, and therefore they were cultured with or without 2.5 μM Ax for an additional 24 h. Aged oocytes treated with Ax showed improved yield and quality of blastocysts as well as recovered expression of maternal genes. Importantly, oxidative stress in aged oocytes was relieved through Ax treatment, based on reduced reactive oxygen species and enhanced glutathione and antioxidant gene expression. Moreover, inhibition in apoptosis and autophagy of aged oocyte by Ax was confirmed through decreased caspase-3, cathepsin B and autophagic activities. Ax could also maintain spindle organization and actin expression, and rescue functional status of organelles including mitochondria, endoplasmic reticulum, Golgi apparatus and lysosomes according to restored fluorescence intensity. In conclusion, Ax might provide an alternative for ameliorating the oocyte quality following aging in vitro, through the mechanisms mediated by its antioxidant properties.

## Introduction

It is generally known that mammalian oocytes are the cornerstone for reproductive biology. The quality of oocytes affects the efficiency of assisted reproductive technologies (ART) such as in vitro fertilization, intracytoplasmic sperm injection and somatic cell nuclear transfer (SCNT), and is essential for successful embryo development and subsequent implantation after transfer^1,2^. However, oocyte quality has been demonstrated to be compromised during either in vivo aging in the oviduct or in vitro aging in the culture^3^. Aging is the process whereby oocytes undergo a series of complex undesirable changes owing to no fertilization or activation in time^4,5^. Accumulating evidences have reported a variety of defects in the aged oocytes, including spindle/chromosome disturbance, organellar dysfunction, zona pellucida hardening, and abnormal regulation of molecular and biochemical events^6,7^. Most importantly, aging process is accompanied by overproduction of reactive oxygen species (ROS) and concomitant depletion of antioxidant system leading to oxidative stress, which impairs the quality and developmental potential of oocytes^8,9^. Up to now many strategies have been developed to protect oocytes against aging in vitro, in order to gain sufficient time for various ART. One of the most effective means is maintain the balance of intracellular redox state in aged oocytes through exogenous supplementation of antioxidant compounds. The typical antioxidants such as melatonin^10–12^, resveratrol^13–15^, N‐acetyl‐L‐cysteine^16^, Coenzyme Q10 ^17^, etc., have been proved to improve the aged oocyte quality.

Astaxanthin (3,3′-dihydroxy-β,β′-carotene-4,4′-dione; Ax) is a natural xanthophyll carotenoid found predominantly in algae, asteroideans, crustacean shells, salmon and other marine organisms^18^. The biological functions of Ax have been studied for many years including anti-cancer, anti-diabetic, anti-inflammatory, anti-obesity and immuno-modulatory, most of which may be mediated by antioxidant activity^19,20^. Ax has a unique molecular structure in the presence of hydroxyl and keto endings on each ionone ring, which is responsible for its excellent antioxidant properties^21,22^. The antioxidant activity of Ax is 10 times more than other carotenoids (canthaxanthin, β-carotene, lutein and zeaxanthin) and 100 times stronger than α-tocopherol^23^. Recently, there has been growing interest in the utilization of Ax in reproductive biology field. Ax has been reported in cattles to ameliorate the developmental defects of embryos impaired by heat stress^24^ or oxidative stress^25^, and contribute to the production of SCNT embryos^26^ or the in vitro growth of oocytes derived from early antral follicles^27^. In humans, treatment with Ax can enhance sperm capacitation^28^ and prevent papillomavirus L1 protein binding in sperm membrane^29^. However, it is unknown whether Ax can protect the oocytes against aging in vitro.

Porcine oocytes provide a more ideal model for the investigation of reproductive biology, as they have developmental and physiological similarities to human oocytes^30^. Therefore, the present study was designed to determine the cytoprotective effect of Ax on porcine oocytes during aging in vitro, and explore the underlying cellular and molecular mechanisms.

## Results

### Ax improves developmental competence of aged porcine oocytes

At the end of in vitro maturation (IVM), cumulus cells were removed by repeated pipetting in Tyrode’s lactate-HEPES-polyvinyl alcohol (TLH-PVA) medium^31^ containing 0.1% (w/v) hyaluronidase, and oocytes with evenly granular cytoplasm and a first polar body were selected for the following experiments. For aging in vitro, oocytes were cultured in porcine zygote medium-3 (PZM-3)^32^ supplemented with or without 2.5 μM Ax (Aged + Ax and Aged groups, respectively) for an additional 24 h at 39 °C in an atmosphere of 5% CO_2_ with saturated humidity. The optimal concentration of Ax was based on our preliminary experiments. The fresh oocytes without any prolonged culture were used as control (Fresh group).

**Figure 1:**
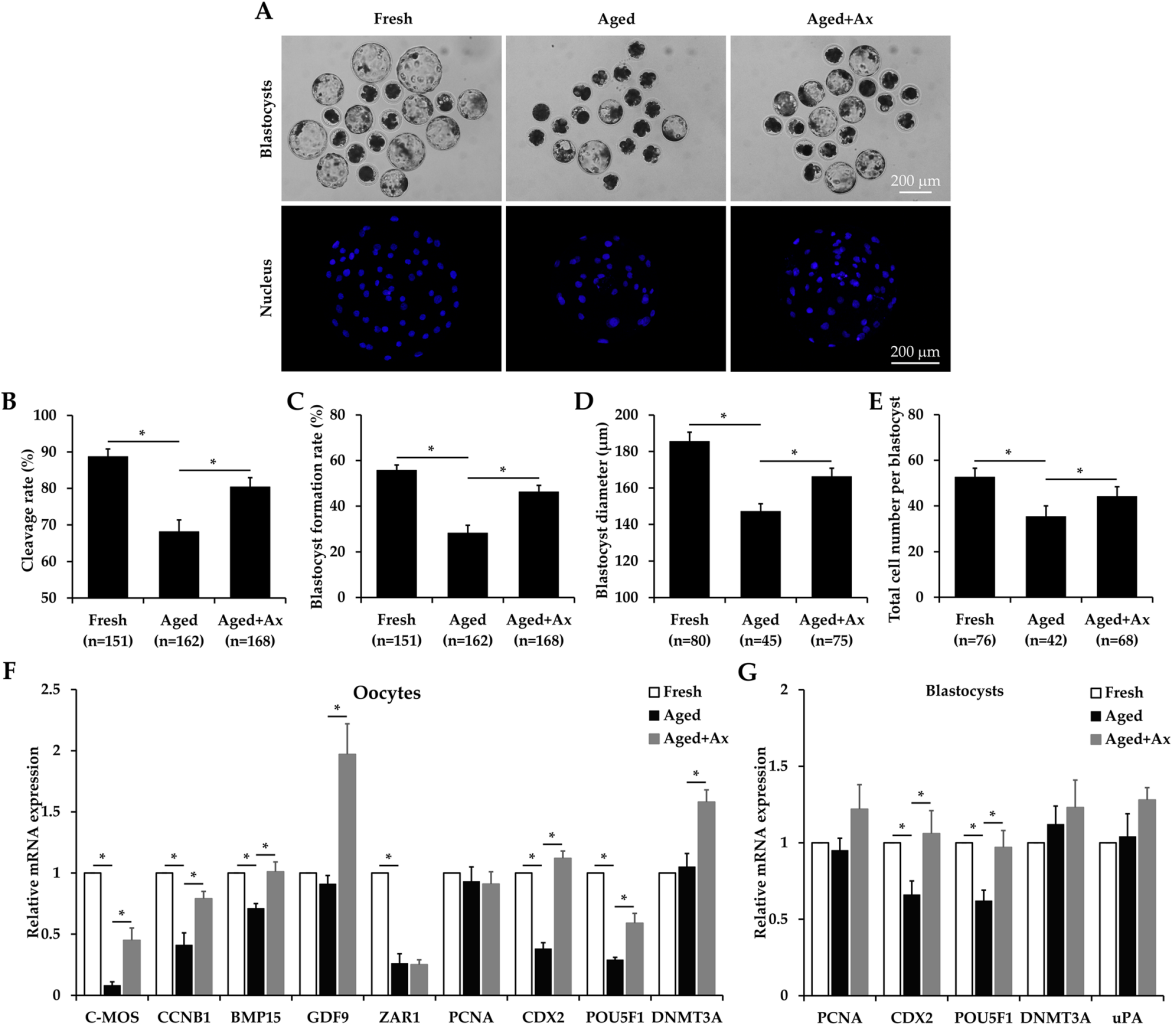
Astaxanthin (Ax) improves developmental competence of aged porcine oocytes. **(A)** Representative images of resultant blastocysts derived from parthenogenetic activation and nucleus stained with Hoechst 33342. **(B,C)** Cleavage and blastocyst formation rates on Days 2 and 6, respectively. **(D,E)** Diameter and total cell number per blastocyst. **(F)** Relative mRNA expression of oocyte maturation and/or embryo development related genes (*C-MOS*, *CCNB1*, *BMP15*, *GDF9*, *ZAR1*, *PCNA*, *CDX2*, *POU5F1* and *DNMT3A*) in oocytes. **(G)** Relative mRNA expression of embryo development related genes (*PCNA*, *CDX2*, *POU5F1*, *DNMT3A* and *uPA*) in resultant blastocysts. Fresh: fresh oocytes; Aged: aged oocytes; Aged + Ax: aged oocytes treated with Ax. Each experiment was independently repeated four times. Values **(B–G)** are presented as the mean ± SEM. * indicate a significant difference between two groups (P < 0.05).

In this experiment, we determined whether Ax could improve the developmental competence of aged oocytes following parthenogenetic activation (PA). As shown in Fig. 1A–C, percentages of cleavage and blastocyst formation were significantly lower in the Aged group (68.2 ± 3.1% and 28.3 ± 3.3%, respectively) than in the Fresh group (88.7 ± 2.1% and 55.7 ± 2.3%, respectively), while the Aged + Ax group increased the cleavage and blastocyst formation rates to 80.4 ± 2.5% and 46.2 ± 2.9%, respectively. There was a significant decrease in blastocyst diameter in the Aged group (147.1 ± 4.2 μm) when compared to the Fresh (185.5 ± 5.1 μm) and Aged + Ax (166.2 ± 4.6 μm) groups (Fig. 1D). Moreover, the Aged group (35.3 ± 4.3) also showed a significant reduction in total cell number per blastocyst than the Fresh (52.6 ± 3.9) and Aged + Ax (44.1 ± 4.5) groups (Fig. 1E).

To further confirm the effects of Ax on the quality of aged oocytes and resultant blastocysts, quantitative real-time reverse transcription polymerase chain reaction (qRT-PCR) was submitted to detect the mRNA expression of oocyte maturation and/or embryo development related genes (*C-MOS*, *CCNB1*, *BMP15*, *GDF9*, *ZAR1*, *PCNA*, *CDX2*, *POU5F1*, *DNMT3A* and *uPA*). As shown in Fig. 1F, the mRNA levels of *C-MOS*, *CCNB1*, *BMP15*, *ZAR1*, *CDX2* and *POU5F1* in oocytes were significantly lower in the Aged group than in the Fresh group, but no differences in gene expression for *GDF9*, *PCNA* and *DNMT3A*. Compared with the Aged group, the Aged + Ax group had significantly up-regulated mRNA expression of these genes, except for *ZAR1* and *PCNA*. For the resultant blastocysts, the mRNA levels of *PCNA*, *CDX2*, *POU5F1*, *DNMT3A* and *uPA* were similar in the Fresh and Aged + Ax groups, but *CDX2* and *POU5F1* showed significantly decreased expression in the Aged group (Fig. 1G).

### Ax alleviates oxidative stress in aged porcine oocytes

**Figure 2:**
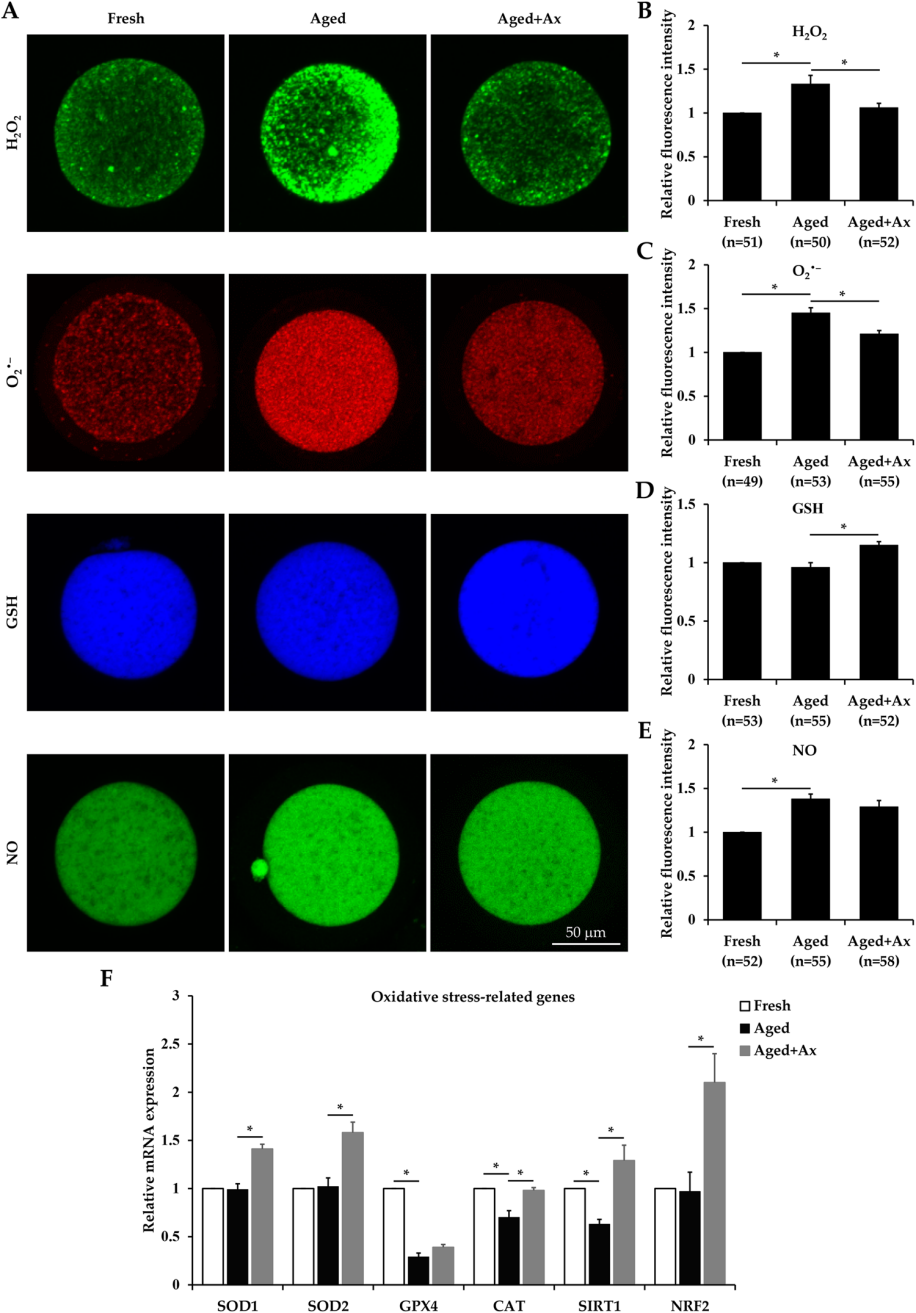
Astaxanthin (Ax) alleviates oxidative stress in aged porcine oocytes. **(A)** Representative images of hydrogen peroxide (H_2_O_2_), superoxide anion (O_2_^•–^), glutathione (GSH) and nitric oxide (NO) in oocytes. **(B–E)** Quantified intracellular levels of H_2_O_2_, O_2_^•–^, GSH and NO by relative fluorescence intensity. **(F)** Relative mRNA expression of oxidative stress related genes (*SOD1*, *SOD2*, *GPX4*, *CAT*, *SIRT1* and *NRF2*) in oocytes. Relative fluorescence intensity for the fresh group was set arbitrarily at 1. Fresh: fresh oocytes; Aged: aged oocytes; Aged + Ax: aged oocytes treated with Ax. Each experiment was independently repeated at least four times. Values **(B–F)** are presented as the mean ± SEM. *indicate a significant difference between two groups (P < 0.05).

For evaluating the efficacy of Ax in reducing oxidative stress, we first measured the levels of intracellular ROS including hydrogen peroxide (H_2_O_2_) and superoxide anion (O_2_^•–^) with the fluorescent probes of CM-H_2_DCFDA and Dihydroethidium, respectively. As shown in Fig. 2A–C, the levels of H_2_O_2_ and O_2_^•–^ were significantly higher in the Aged group than in the Fresh group. In the Aged + Ax group, the two values decreased significantly compared to these in the Aged group. Reduced glutathione (GSH) is an important intracellular antioxidant component for counteraction of oxidative stress injury, and so GSH level was subsequently detected by ThiolTracker Violet probe. Surprisingly, intracellular GSH level did not differ between the Fresh and Aged groups, and was significantly lower compared to that in the Aged + Ax group (Fig. 2A,D). In addition, we also examined intracellular nitric oxide (NO) production by DAF-FM Diacetate, because of its responsibility for oxidative stress. Both the Aged and Aged + Ax groups showed similar level of NO, but significantly higher compared to the fresh group (Fig. 2A,E).

Finally, we determined the mRNA expression of antioxidant and oxidative stress related genes (*SOD1*, *SOD2*, *GPX4*, *CAT*, *SIRT1* and *NRF2*) by qRT-PCR analysis. Compared to the Fresh group, the Aged group showed significantly decreased mRNA levels of *GPX4*, *CAT* and *SIRT1*, but had a similar expression for *SOD1*, *SOD2* and *NRF2* genes (Fig. 2F). As expected, the mRNA levels of these genes except for *GPX4* were significantly increased in the Aged + Ax group than in the Aged group (Fig. 2F).

### Ax inhibits apoptosis and autophagy in aged porcine oocytes

**Figure 3:**
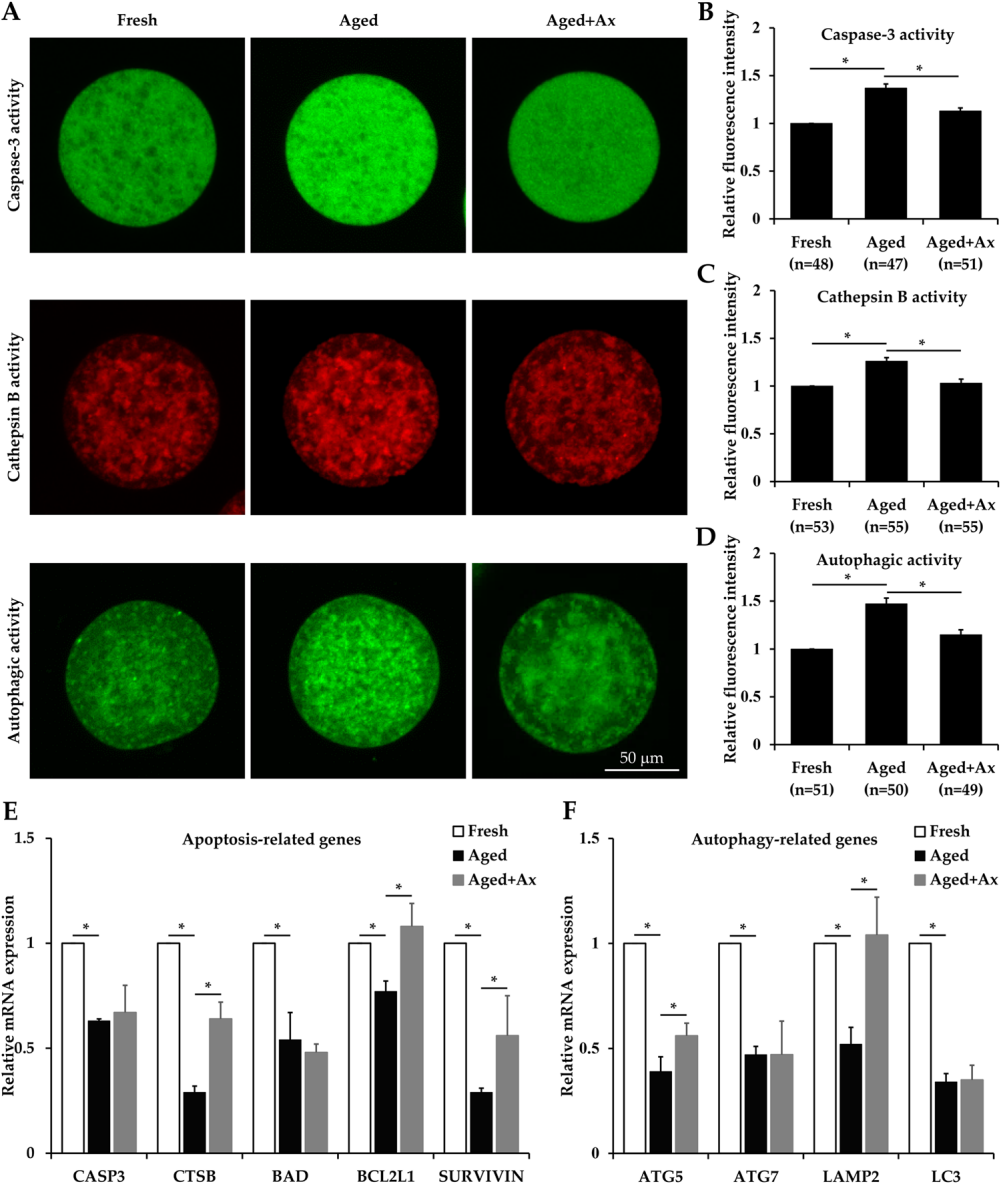
Astaxanthin (Ax) inhibits apoptosis and autophagy in aged porcine oocytes. **(A)** Representative images of caspase-3 activity, cathepsin B activity and autophagic activity in oocytes. **(B–D)** Quantified intracellular activities of caspase-3, cathepsin B and autophagy by relative fluorescence intensity. **(E)** Relative mRNA expression of apoptosis related genes (*CASP3*, *CSTB*, *BAD*, *BCL2L1* and *SURVIVIN*) in oocytes. **(F)** Relative mRNA expression of autophagy related genes (*ATG5*, *ATG7*, *LAMP2* and *LC3*) in oocytes. Relative fluorescence intensity for the fresh group was set arbitrarily at 1. Fresh: fresh oocytes; Aged: aged oocytes; Aged + Ax: aged oocytes treated with Ax. Each experiment was independently repeated at least four times. Values **(B–F)** are presented as the mean ± SEM. *indicate a significant difference between two groups (P < 0.05).

It is generally known that a series of cellular apoptotic events occur during oocyte aging in vitro. Caspase-3 is a key mediator of apoptosis; therefore its activity was measured using a CaspGLOW Fluorescein Active Caspase-3 Staining Kit. As shown in Fig. 3A,B, the caspase-3 activity in the Aged group was significantly higher than that in the Fresh group, and the Aged + Ax group significantly decreased this level. In addition, cathepsin B as a lysosomal cysteine protease ascribes a proapoptotic function. We next quantified the cathepsin B activity by Magic Red Cathepsin B Assay Kit. Notably, the cathepsin B activity was significantly decreased in the Aged + Ax group compared with the Aged group, similar to the Fresh group (Fig. 3A,C). Furthermore, the mRNA expression of apoptosis related genes (*CASP3*, *CSTB*, *BAD*, *BCL2L1* and *SURVIVIN*) were also analyzed. qRT-PCR results showed that the mRNA levels of these genes in the Aged group were significantly decreased than those in the Fresh group, and the Aged + Ax group had significantly increased mRNA levels for *CSTB*, *BCL2L1* and *SURVIVIN* when compared with the Aged group (Fig. 3E).

On the other hand, autophagy is reported to be involved in the process of oocyte aging. As Ax can modulate autophagy by regulating signaling pathways, we examined whether it continued to inhibit the autophagy in aged oocytes. Firstly, the autophagic activity was measured by a CYTO-ID Autophagy Detection Kit. As shown in Fig. 3A,D, the Aged group presented a significantly higher autophagic activity compared to the Fresh group, and the level was remarkably reduced in the Aged + Ax group. Moreover, the autophagy related genes (*ATG5*, *ATG7*, *LAMP2* and *LC3*) of the Aged group also showed significantly lower mRNA expression compared to those of the Fresh group. Expectedly, the mRNA levels of *ATG5* and *LAMP2* were significantly higher in the Aged + Ax group than in the Aged group (Fig. 3F).

### Ax rescues spindle defects and actin filaments in aged porcine oocytes

**Figure 4:**
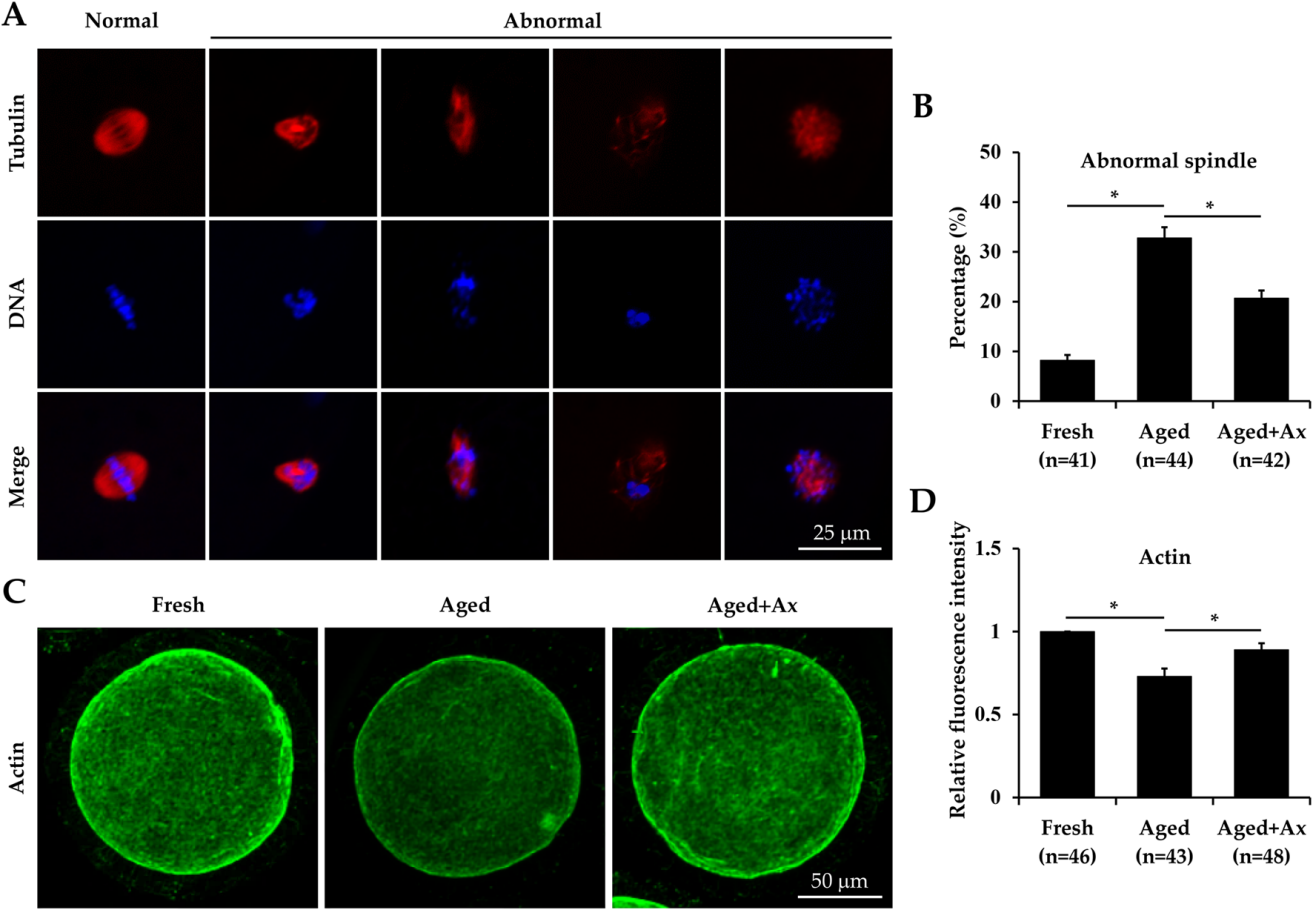
Astaxanthin (Ax) rescues spindle defects and actin filaments in aged porcine oocytes. **(A)** Representative images of spindle morphologies in oocytes. **(B)** The percentage of oocytes with abnormal spindles. **(C)** Representative images of actin localization in oocytes. **(D)** Quantified intracellular actin expression by relative fluorescence intensity. Relative fluorescence intensity for the fresh group was set arbitrarily at 1. Fresh: fresh oocytes; Aged: aged oocytes; Aged + Ax: aged oocytes treated with Ax. Each experiment was independently repeated at least four times. Values **(B,D)** are presented as the mean ± SEM. *indicate a significant difference between two groups (P < 0.05).

Cytoskeleton abnormality has been confirmed during oocyte aging in vitro. So, we detected spindle morphology and actin expression of the aged oocytes following Ax treatment. As shown in Fig. 4A, most fresh oocytes displayed a typical barrel-shaped spindle with a well-aligned chromosome. However, various forms of abnormal spindles such as condensation, elongation, dispersal, or disruption occurred in aged oocytes, and chromosome failed to align at the metaphase plate. The percentage of oocytes with abnormal spindles was significantly lower in the Aged + Ax group (20.7 ± 1.5%) than in the Aged group (32.8 ± 2.1%), and was still significantly higher compared to the Fresh group (8.2 ± 1.1%) (Fig. 4B). Actin filaments in oocytes were distributed mainly in the cortical region with accumulating strong signals (Fig. 4C). Moreover, the relative fluorescence intensity of actin filaments was significantly decreased in the Aged group compared with the Fresh group, and could be markedly increased in the Aged + Ax group (Fig. 4D).

### Ax maintains organelle functional status in aged porcine oocytes

**Figure 5:**
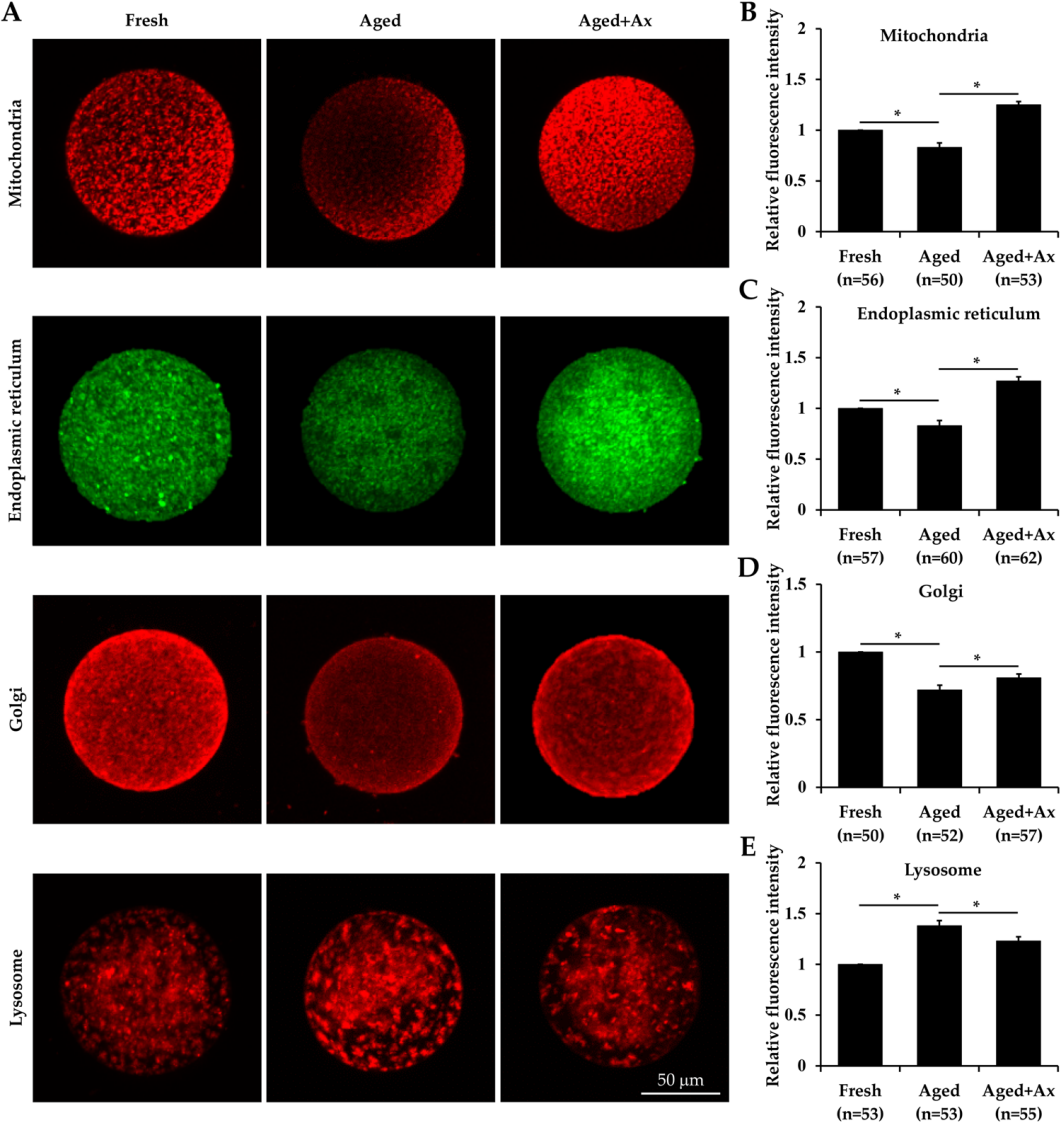
Astaxanthin (Ax) maintains organelle functional status in aged porcine oocytes. **(A)** Representative images of mitochondria, endoplasmic reticulum (ER), Golgi apparatus and lysosomes in oocytes. **(B–E)** Intracellular relative fluorescence intensity of mitochondria, ER, Golgi apparatus and lysosomes. Relative fluorescence intensity for the fresh group was set arbitrarily at 1. Fresh: fresh oocytes; Aged: aged oocytes; Aged + Ax: aged oocytes treated with Ax. Each experiment was independently repeated at least four times. Values **(B–E)** are presented as the mean ± SEM. *indicate a significant difference between two groups (P < 0.05).

Aging often results in disturbances in morphology and function of intracellular organelles, thus impairing oocyte quality. Next, we intended to explore the beneficial effects of Ax on organelles in the aged oocytes, including mitochondria, endoplasmic reticulum (ER), Golgi apparatus and lysosomes. These organelles were labeled through respective fluorescent probes. The different values of fluorescence intensity can represent the distinction in organelle functional status. The relative fluorescence intensity showed that the values of mitochondria, ER and Golgi apparatus were significantly decreased in the Aged group compared with the Fresh group; meanwhile, these values were considerably improved in the Aged + Ax group (Fig. 5A–D). On the other hand, the relative fluorescence intensity of lysosomes in the Aged group were significantly higher than that in both the Fresh and Aged + Ax groups (Fig. 5A,E).

## Discussion

Oxidative stress results from an imbalance of oxidant and antioxidant, which triggers a cascade of unfavorable factors to directly affect multiple aspects of biochemistry and functionality in the aged oocytes^33,34^. Moreover, the antioxidant Ax has been proved to alleviate oxidative stress in various cell types, including sperms, oocytes and embryos. In the present study, we revealed that Ax was able to delay oocyte aging in vitro, owing to possible mechanisms mediated by reducing oxidative stress.

It is well known that aged oocytes exhibit a very poor ability to fertilization and embryo development, and the quality of resultant blastocysts is also compromised^35,36^. For identifying the beneficial effects of Ax on aged oocytes, the most straightforward way is to evaluate their developmental competence. In this study, the blastocyst yield, and diameter and total cell number per blastocyst were improved when the oocytes were treated with Ax during aging in vitro, suggesting that Ax could enhance the capacity of embryos to develop to the blastocyst stage and the embryo quality. As we know, the maternal gene in oocytes is required for preimplantation embryo development^37^. Differences in maternal gene expression lead to the lower developmental potential of aged oocytes. For instance, ZAR1 plays essential role in transition from oocyte to embryo^38^, and its expression in this study was found to be reduced in aged oocytes. Furthermore, Ax treatment was helpful to maintain the maternal gene expression for *C-MOS*, *CCNB1*, *BMP15*, *CDX2* and *POU5F1* in aged oocytes. Our results also showed that the mRNA levels of *CDX2* and *POU5F1* genes were also increased in resultant blastocysts from Ax-treated aged oocytes. These two genes are required for differentiation of trophectoderm during early embryo development^39^. Therefore, the above findings provided the most direct proof of the beneficial effects of Ax for aged oocytes.

Currently, the adverse effects of oxidative stress occurred in aged oocytes have been well documented. The main reason for the oxidative stress is that aged oocytes often exhibit high levels of intracellular ROS. Ax acts as a scavenger of free radicals working on singlet oxygen, H_2_O_2_, O_2_^•–^, hydroxyl radical and reactive nitrogen species^40^. In the present study, Ax could effectively reduce the levels of H_2_O_2_ and O_2_^•–^ in aged oocytes. In addition, we found that an accumulation of NO occurred in aged oocytes, but Ax treatment did not decrease its level, unlike in other cell types. GSH as an endogenous antioxidant not only maintain the intracellular redox balance, but also is an indicator of cytoplasmic maturation to reflect the oocyte fertilization and subsequent development^41^. There was no reduction of GSH content for aged oocytes in this study, similar to previous reports^42,43^; however, several studies have showed the aged oocytes with lower level of GSH^44,45^. Moreover, the aged oocytes treated with Ax exhibited an increase in intracellular GSH content, which is similar to the result of antioxidant hesperetin treatment^42^. The genes related to antioxidation pathway are frequently detected to evaluate the oxidative stress in aged oocytes, but different results have been obtained due to different experimental conditions. It has been confirmed that Ax can play a major role in the modulation of redox signaling pathways such as SIRT1 and NRF2, suppressing oxidative damage from various stress conditions^46^. We found that the oocyte aging in vitro resulted in decreased mRNA levels of *GPX4*, *CAT* and *SIRT1*, but did not affect the *SOD1*, *SOD2* and *NRF2* expression. These genes except for *GPX4* in aged oocytes showed increased mRNA expression after treatment with Ax. In a word, Ax exhibited a better radical scavenging capacity to attenuate oxidative stress during oocyte aging in vitro.

Oxidative stress can lead to apoptosis through numerous mechanisms and then cell death. The degree of apoptosis has been confirmed to be increased in aged oocytes^9^. Caspase-3 acts as a key promoter of apoptosis, because all apoptotic pathways eventually converge on the caspase-3 activation^47^. Cathepsin B is closely associated with the process of apoptosis by activating caspases^48^. Similar to previous studies^44,49^, we also observed a significant rise in the activities of caspase-3 and cathepsin B during oocyte aging in vitro. In addition, Ax is known to be able to regulate the mitochondria-mediated apoptosis probably caused by signaling pathways such as NF-κB and Wnt/β-catenin, involving a downregulation of pro-apoptotic proteins (eg. Bax and cleaved caspase-3) and an upregulation of anti-apoptotic proteins (eg. Bcl-2 and survivin)^50,51^. Treatment with Ax has also been reported to decrease the cathepsin B activity^27^. The present study found that the activities of these two proteases were efficiently inhibited in aged oocytes following Ax treatment. On the other hand, autophagy is induced to enable cellular survival under different stress conditions, but it also plays the functions in cell death^52^. It has been reported that the aging process of oocytes is accompanied by autophagy activation^53^. Several studies have demonstrated the potential of Ax in regulating autophagy^54^. Expectantly, Ax could meliorate the autophagic activity during oocyte aging in vitro. Our study also found that the aged oocytes treated with Ax exhibited a relatively stable expression for some genes related to apoptosis and autophagy, including *CSTB*, *BCL2L1*, *SURVIVIN*, *ATG5* and *LAMP2*. Thus, the above results indicated the effective inhibition of Ax on apoptosis and autophagy in aged oocytes.

The spindle as an important structure for accurate chromosomal distribution, is correlated with coordination interaction between the actin and microtubule cytoskeleton. The aged oocytes often present with spindle disruption and actin reduction^55^. In the present study, Ax treatment attenuated the spindle abnormality and restored the actin expression for aged oocytes, indicating its protection on cytoskeletal stability. On the other hand, cytoplasmic maturation of oocytes is dependent on the dynamic distribution and functional integrity of organelles^56^. The functional state changes in any kind of organelles including mitochondria, ER, Golgi apparatus and lysosomes can affect oocyte quality and early embryonic development. These oocyte organelles are especially vulnerable to oxidative stress. Still oocyte aging in vitro results in the abnormal distribution and function disorder of cytoplasmic organelles. For example, the most widely studied organelle is mitochondria, with confirmed dysfunction in aged oocytes such as low membrane potential, reduced ATP content and mutated mitochondrial DNA^57,58^. The aged porcine oocytes has been reported to exhibit disordered ER clusters and impaired ER calcium homeostasis^59^. In the present study, oocyte organelles were label with specific fluorescent probes, and the change in quantification of fluorescence intensity might reflect organelle status. The mitochondria, ER and Golgi apparatus in aged oocytes showed a decrease in the fluorescence intensity, indicating their functional decline. In addition, the fluorescence intensity of lysosomes was elevated in aged oocytes, which seemed to be associated with increased autophagic activity as described above. Because autophagy is a highly conserved lysosome-dependent pathway^60^. We further found that Ax was able to effectively recover the fluorescence intensity of these organelles in aged oocytes, indicating powerful amelioration of dysfunctional organelles. The beneficial effects of Ax on various subcellular organelles may be attributed to its antioxidant properties, role as signaling molecule and gene regulator that are involved in the regulation of organellar functions such as mitochondrial redox state and ER stress^61,62^. In brief, these results suggested the positive effects of Ax treatment on cytoplasmic maturation for aged oocytes from the perspective of cytoskeleton and organelle status.

Taken all together, our results demonstrated that Ax treatment could ameliorate the quality and developmental competence of aged porcine oocytes, which was probably at least partly attributable to the alleviation of oxidative stress, apoptosis and autophagy, and the stabilization of cytoskeleton and organelle status. This study provides new insights for the protective role of Ax against oocyte aging in vitro, and so will be quite helpful for ART.

## Materials and methods

### Reagents

All chemicals used in this study were purchased from Sigma‐Aldrich Chemical Company (Shanghai, China), unless otherwise stated. Medium 199 (TCM-199), Dulbecco’s PBS (DPBS), CM-H_2_DCFDA, Dihydroethidium, ThiolTracker Violet, DAF-FM Diacetate, MitoTracker Red CMXRos, ER-Tracker Green, LysoTracker Red were obtained from ThermoFisher Scientific (Shanghai, China).

### Oocyte collection and IVM

The procedures and medium used for oocyte collection and IVM were essentially as described by us previously^63^. The porcine ovaries collected from pre-pubertal gilts at a local slaughterhouse (Shennong abattoir; Kunming, China) and transported to our laboratory at 35–37 °C in 0.9% NaCl (w/v) containing 75 mg/L penicillin G potassium and 50 mg/L streptomycin sulfate. Follicular fluid from the follicles (3–8 mm in diameter) was aspirated using a disposable syringe with an 18-gauge needle and placed in a conical tube to allow cumulus‐oocyte complexes (COCs) to settle at the bottom. The precipitates containing COCs were washed twice in TLH-PVA medium and transferred to a petri dish. The COCs with compact cumulus cells and uniform cytoplasm were picked under a stereomicroscope (Olympus, Tokyo, Japan) and washed three times with IVM medium. Then, a group of 50–70 COCs was placed in each well of a 24-well plate (Costar, Corning, NY, USA) containing 500 μL IVM medium for 42–44 h at 39 °C in an atmosphere of 5% CO_2_ with saturated humidity. The IVM medium was TCM-199 supplemented with 10% (v/v) porcine follicular fluid, 3.05 mM D-glucose, 0.57 mM cysteine, 0.91 mM sodium pyruvate, 10 ng/mL epidermal growth factor, 0.5 μg/mL each follicle-stimulating hormone and luteinizing hormone.

### PA and embryo culture

Oocyte activation and embryo culture were conducted as previously reported^64^. Those chosen oocytes were placed between two wires of a microslide 0.5 mm fusion chamber (Model 450; BTX, SanDiego, CA, USA) covered with activation medium (0.28 M mannitol, 0.1 mM MgSO_4_, 0.05 mM CaCl_2_ and 0.5 mM HEPES) and then stimulated with a direct current pulse of 1.3 kV/cm for 80 μs using a BLS CF-150/B cell fusion machine (BLS, Budapest, Hungary). The oocytes were washed three times in PZM-3 supplemented with 5 μg/mL cytochalasin B and 10 μg/mL cycloheximide and incubated in the same medium for 4 h. Finally, they were cultured in PZM-3 at 39 °C in a humidified atmosphere with 5% CO_2_. Percentages of cleavage and blastocyst formation were recorded on Days 2 and 6, respectively (day of PA was designated Day 0). Blastocyst diameter was measured using an inverted microscope (IX71; Olympus, Tokyo, Japan) equipped with a CCD camera. Total cell number per blastocyst was examined by 10 μg/mL Hoechst 33,342 diluted in DPBS containing 0.3% (w/v) polyvinyl alcohol (DPBS-PVA).

### Measurement of oxidative stress

To measure the levels of intracellular H_2_O_2_, O_2_^•–^, GSH and NO, oocytes were incubated for 30 min at 39 °C in DPBS-PVA supplemented with 10 μM CM-H_2_DCFDA, 10 μM Dihydroethidium, 10 μM ThiolTracker Violet or 5 μM DAF-FM Diacetate. After washing three times in DPBS-PVA, the stained oocytes placed into 100 μL DPBS-PVA on a glass bottom cell culture dish and observed using a confocal laser-scanning microscope (Nikon A1, Nikon, Tokyo, Japan) with excitation wavelength at 405 nm (blue, for imaging GSH), 488 nm (green, for imaging H_2_O_2_ and NO) or 535 nm (red, for imaging O_2_^•–^).

### Assessment of apoptosis and autophagy

For assessing caspase-3 and cathepsin B activities, oocytes were incubated with FITC-DEVD-FMK (1:300 in DPBS-PVA; BioVision, Mountain View, CA, USA) or MR-RR2 (1:250 in DPBS-PVA; Immunochemistry, Bloomington, MN, USA) for 30 min at 39 °C in the dark, washed three times in DPBS-PVA, and then determined immediately under a confocal laser-scanning microscope with excitation wavelength at 488 nm (green, for imaging caspase-3 activity) or 535 nm (red, for imaging cathepsin B activity). Autophagic activity detection was performed using Cyto-ID Green (1:1000 in DPBS-PVA; Enzo Life Sciences, Farmingdale, NY, USA) to stain autophagic compartments, with all other steps performed as above.

### Immunofluorescence staining

At room temperature, oocytes were fixed in 4% (w/v) paraformaldehyde for 30 min, permeabilized with 0.1% Triton X-100 for 30 min, and then incubated for 1 h in the dark with anti-α-tubulin-TRITC antibody (1:100 in dilution, Beyotime, Jiangsu, China) or phalloidin-FITC (1:200 in dilution, Beyotime, Jiangsu, China) diluted in DPBS containing 5% BSA and 0.1% Triton X-100. After washing in 0.1% Triton X-100, these oocytes were stained with 10 μg/mL Hoechst 33342 for 10 min to label the DNA. Finally, the stained oocytes were washed three times with DPBS-PVA, mounted on glass slides and imaged using a confocal laser-scanning microscope.

### Staining of intracellular organelles

To probe mitochondria, ER and lysosomes, oocytes were stained with 200 nM MitoTracker Red CMXRos, 200 nM ER-Tracker Green, or 50 nM LysoTracker Red for 30 min at 39 °C in the dark, washed three times in DPBS-PVA and then observed under a confocal laser-scanning microscope with excitation wavelength at 535 nm (red, for imaging mitochondria and lysosomes) or 488 nm (green, for imaging ER). For staining Golgi apparatus, oocytes were incubated in Golgi-Tracker Red (1:100 in dilution, Beyotime, Jiangsu, China) for 30 min at 4 °C in the dark, washed three times with cooled DPBS-PVA and then cultured for 30 min at 39 °C. Subsequently, they were examined using a confocal laser-scanning microscope with excitation wavelength at 535 nm (red, for imaging Golgi apparatus).

### qRT-PCR

Total complementary DNA was isolated from 15 oocytes or 5 blastocysts using a TransScript-Uni Cell to cDNA Synthesis SuperMix for qPCR (TransGen Biotech, Beijing, China) according to the manufacturer’s protocol, and stored at − 20 °C until analyzed. qRT-PCR was conducted using a CFX Real-Time PCR Detection System (Bio-Rad, Hercules, CA, USA) with Fast qPCR Mix (Tsingke, Beijing, China). Reaction conditions were 95 ˚C for 1 min, followed by 40 cycles of 95 ˚C for 10 s and 60 ˚C for 15 s. Four replicates were done for each reaction. Relative expression of target genes was quantified by the 2^−ΔΔCT^ method after normalization to GAPDH (an internal control gene) mRNA abundance. Primer sequences used in this experiment are listed in Supplementary Table S1.

### Statistical analysis

Images of oocytes stained in the same dye were captured with the same scan settings. The average value of fluorescence intensity in each oocyte was quantified after deduction of the background fluorescence through ImageJ software (National Institutes of Health, Bethesda, MD, USA). Fluorescence values for both Aged and Aged + Ax groups were determined relative to that of the Fresh group, and Fresh group value was randomly set to 1. Data were evaluated by ANOVA (SPSS 20.0 software, SPSS Inc., Chicago, IL, USA) followed by Student–Newman–Keuls test for multiple comparisons, and percentage data such as cleavage and blastocyte formation rates were arcsine transformed prior to analysis. All results were presented as least squares mean ± SEM values, and the P-value < 0.05 was defined as significant.

## Supplementary information

Supplementary Table S1.

## Data Availability

The datasets generated during and/or analysed during the current study are available from the corresponding author on reasonable request.
