# Supplementary material for: Inhibitory effects of astaxanthin on postovulatory porcine oocyte aging in vitro
Source: Sci Rep. 2020 Nov 19;10:20217. doi: 10.1038/s41598-020-77359-6 (PMC7677382; doi:10.1038/s41598-020-77359-6)
Supplement: Supplementary file 1 — Supplementary Table S1. [file 41598_2020_77359_MOESM1_ESM.docx]

**Inhibitory effects of** **astaxanthin on postovulatory porcine oocyte aging *in vitro***

Bao-Yu Jia^1,⸸^, De-Cai Xiang^2,⸸^, Qing-Yong Shao^2^, Bin Zhang^2^, Shao-Na Liu^2^, Qiong-Hua Hong^2^, Guo-Bo Quan^2*^ & Guo-Quan Wu^2*^

^1^College of Veterinary Medicine, Yunnan Agricultural University, Kunming, Yunnan 650201, China.

^2^Yunnan Provincial Engineering Laboratory of Animal Genetic Resource Conservation and Germplasm Enhancement, Yunnan Animal Science and Veterinary Institute, Kunming, Yunnan, 650224, China.

^*^Corresponding author.

E-mail address: waltq20020109@163.com (G.-B.Q.) and wuguoquan1982@163.com (G.-Q.W.).

^⸸^These authors contributed equally to this work: Bao-Yu Jia and De-Cai Xiang.

**Supplementary Table S1.** Primers used for gene expression analysis.

| Genes | Primer sequences | Product size (bp) | GenBank accession number |
| --- | --- | --- | --- |
| *ATG5* | F: ACCACTGCTTTCCTCCACTG | 200 | NM_001037152 |
|  | R: CCATGGTCACCTCAGGAAAT |  |  |
| *ATG7* | F: GAACGGGAAGGATTTAATTT | 70 | NM_001190285.1 |
|  | R: CAGTCAAGTCCTCCAAGAAG |  |  |
| *BAD* | F: GGACTGAGGATGAGGAGCTC | 118 | XM_021082883.1 |
|  | R: CCCTGGAACTCGTCACTCAT |  |  |
| *BCL2L1* | F: GGAGCTGGTGGTTGACTTTC | 137 | XM_021099593.1 |
|  | R: TGATGGCACTAGGGGTTTCC |  |  |
| *BMP15* | F: CGCCATCAACTTCACCTAGC | 154 | NM_001005155.1 |
|  | R: GTTGCGTGACATCCATCTCC |  |  |
| *CASP3* | F: GAGGCAGACTTCTTGTATGC | 237 | NM_214131 |
|  | R: CATGGACACAATACATGGAA |  |  |
| *CAT* | F: ACATGGTCTGGGACTTCTGG | 100 | NM_214301 |
|  | R: TCATGTGCCTGTGTCCATCT |  |  |
| *CCNB1* | F: CCTACTGGGTCGTGAAGTCA | 186 | L48205.1 |
|  | R: AACATGGCAGTGACACCAAC |  |  |
| *CDX2* | F: TCCTTTAGTGCTGGCAGGAA | 108 | NM_001278769.1 |
|  | R: CAACCAGTCGATGCATCCTG |  |  |
| *C-MOS* | F: TACACCAGGTCATCTACGGC | 173 | NM_001113219.1 |
|  | R: CAGAATGTTCGCTGGCTTCA |  |  |
| *CTSB* | F: CTCTAGGAACGAGAAGGAGAT | 99 | NM_001097458.1 |
|  | R: CCAGACTTATACTGCAGGAAG |  |  |
| *DNMT3A* | F: CATCCGGGTGCTGTCTCTAT | 160 | XM_005662686.2 |
|  | R: GTCCCCGACGTACATGATCT |  |  |
| *GDF9* | F: GATTGATGTGACGGCCATCC | 183 | NM_001001909.1 |
|  | R: TGTGTGCTTGTGTCGTTCAG |  |  |
| *GPX4* | F: ATTCTCAGCCAAGGACATCG | 93 | NM_214407.1 |
|  | R: CCTCATTGAGAGGCCACATT |  |  |
| *LAMP2* | F: CACCCACTCCAAAGGAAAAA | 246 | XM_001926458 |
|  | R: GGTTGTCGTTTTTCACAGCA |  |  |
| *LC3* | F: CCGAACCTTCGAACAGAGAG | 206 | NM_001190290 |
|  | R: AGGCTTGGTTAGCATTGAGC |  |  |
| *NRF2* | F: CATAGCAGAGCCCAGTACCA | 134 | XM_013984303 |
|  | R: CACGGTGGTCTTGGTTGAAG |  |  |
| *PCNA* | F: AGAGGAGGAAGCAGTTACCAT | 104 | NM_001291925.1 |
|  | R: CTGTAGGAGAGAGTGGAGTGG |  |  |
| *POU5F1* | F: GCGGACAAGTATCGAGAACC | 200 | XM_021097869.1 |
|  | R: CCTCAAAATCCTCTCGTTGC |  |  |
| *SIRT1* | F: TCACTGTGGTAGAGCTTGCA | 112 | NM_001145750 |
|  | R: ACGCTCCCCAAGTTGAAGTA |  |  |
| *SOD1* | F: TCCATGTCCATCAGTTTGGA | 131 | NM_001190422.1 |
|  | R: AGTCACATTGCCCAGGTCTC |  |  |
| *SOD2* | F: AAGCCATCAAACGCGACTTT | 107 | NM_214127.2 |
|  | R: CCTTGTTGAAACCGAGCCAA |  |  |
| *SURVIVIN* | F: CTGCAAAGAAAGTCCGCTGT | 181 | NM_214141 |
|  | R: TTCAAAAGGCTGGCGTTCTC |  |  |
| *uPA* | F: GCTGTCTGAATGGAGGAAAATG | 116 | NM_213945 |
|  | R: GCAGGTTTGCGATGTGTCTATC |  |  |
| *ZAR1* | F: GCGCTTCCAGTTCTTAGAGC | 225 | NM_001129956.1 |
|  | R: ACGTGGCGAAGTTTTACTGG |  |  |
| *GAPDH* | F: GACCCCTTCATTGACCTCCA | 131 | NM_001206359.1 |
|  | R: TGGAAGATGGTGATGGCCTT |  |  |

F = primer forward; R = primer reverse.
